# Review on the extraction of bioactive compounds and characterization of fruit industry by-products

**DOI:** 10.1186/s40643-022-00498-3

**Published:** 2022-02-18

**Authors:** Abhipriya Patra, S. Abdullah, Rama Chandra Pradhan

**Affiliations:** Department of Food Process Engineering, National Institute of Technology, Rourkela, Odisha 769008 India

**Keywords:** Fruit by-products, Extraction technologies, Biowaste valorisation, Characterization, Bioactive compounds

## Abstract

The by-products produced from fruit processing industries could be a potential hazard to environmental pollution. However, these by-products contain several biologically active molecules (essential fatty acid, phenolic compounds, flavonoids, coloring pigments, pectin, proteins, dietary fibers, and vitamins), which can be utilized for various applications in the food, pharmaceutical, cosmetic and textile industries. Nevertheless, during extraction, these bioactive compounds' recovery must be maximized using proper extraction technologies, keeping both economy and environment under consideration. In addition, the characteristics of the extract obtained from those by-products depend mainly on the parameters considered during the extraction process. In this review, an overview of different technologies used to extract bioactive compounds from fruit industry by-products such as seeds and peels has been briefly discussed, along with their mechanisms, process, advantages, disadvantages, and process parameters. In addition, the characteristics of the extracted bioactive compounds have also been briefly discussed in this review.

**Graphical Abstract:**

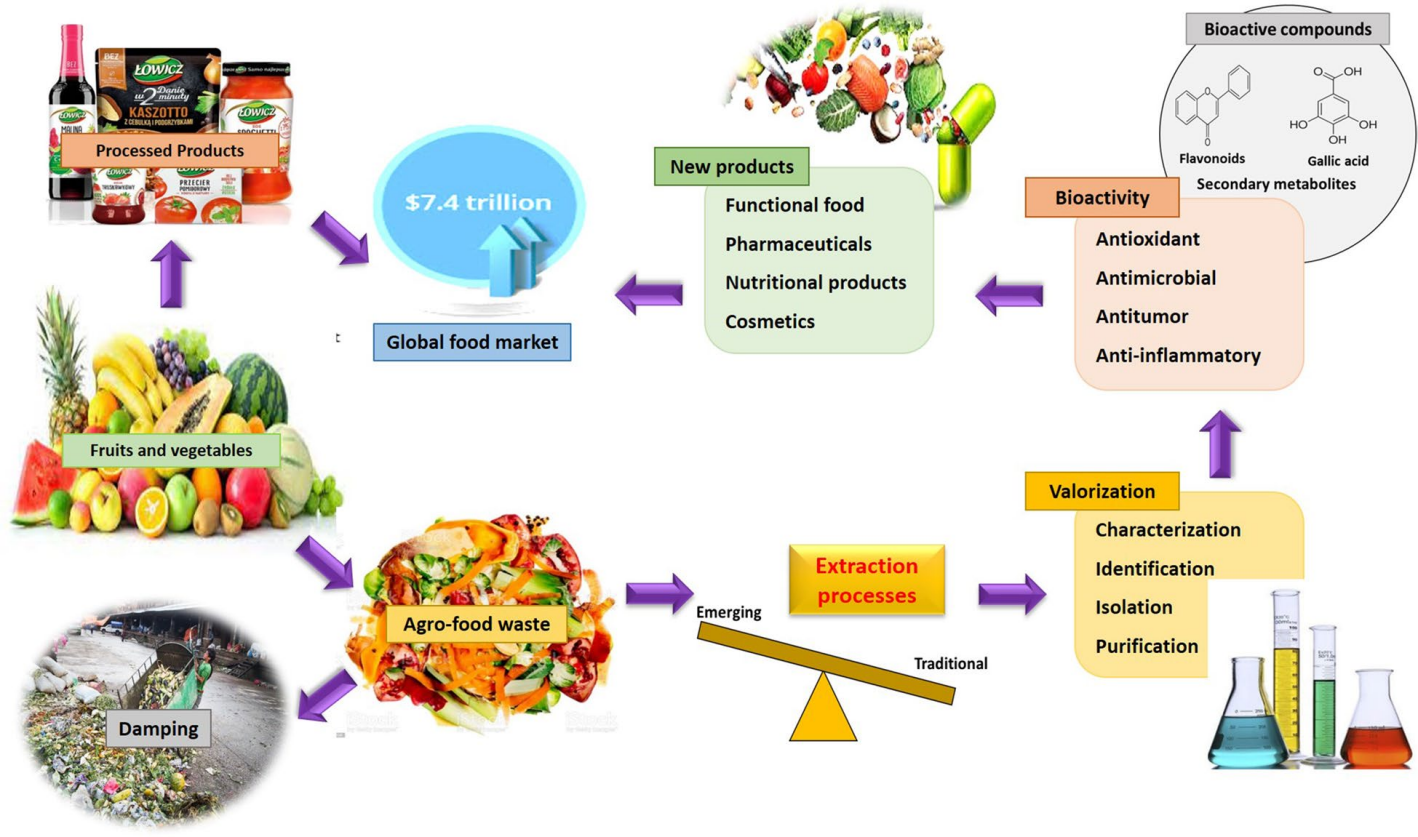

## Statement of novelty

The by-products management of fruit and vegetable industries are becoming a universal concern. The problems can be lessened by applying the extraction conception which means the recovery of bioactive compounds, vitamins, fatty acids, minerals and polysaccharides from the bio-residues, such as seeds and peels. Several studies in the literature have reviewed the different extraction technologies for the valorization of waste. In addition, the technologies are compared with their acceptance of the industrial level. However, the review has also explored the different characterization processes of extracted bioactive compounds. Moreover, integration between two or more extraction technologies for the recovery of bioactive compounds have been enlightened in this review. Overall, this study describes the valorization of waste produced from industries by extracting bioactive compounds with help of different traditional and novel extraction technologies.

## Introduction

Fruits are the potential source of various nutrients, such as macronutrients (carbohydrate, lipids, protein) and micronutrients (vitamins, minerals, bioactive compounds). Hence, these are converted into different processed products, such as juice, jam, jelly, beer, wine, sauces, pickles, and several other products. The global production of fruits is about 503.3 million metric tons; however, only 1.4 million metric tons are commercially processed into different products. During processing, the industries produce and discard at least 25–30% of each fruit as waste products or by-products (Mahato et al. 2019). The significant by-products from fruit processing industries include peel/skin, seeds, leaves, tubers, roots, and pomace. Discarding these by-products harms the environment and generates an economic burden to the concerned industries (Altemimi et al. 2017a; Chemat et al. 2012; Kapoor et al. 2020).

On the other hand, these by-products are an excellent source of bioactive compounds, such as phenolic compounds (phenolic acid, carotenoids, flavonoids), bioactive proteins (peptide isolate, amino acids), fatty acids, fibres, and so on. For instance, the seeds of fruits are a good source of essential oils, phytochemicals, and phytosterols. Similarly, the peels contain pectin, valuable fibers, and minerals (Marić et al. 2018; Mena-García et al. 2019). These bioactive compounds can be extracted from the by-products using different technologies and can be utilized to develop various valorized products, including functional foods or dietary supplements. In addition, in this way, the disposal of waste to the environment can be minimized.

**Fig. 1:**
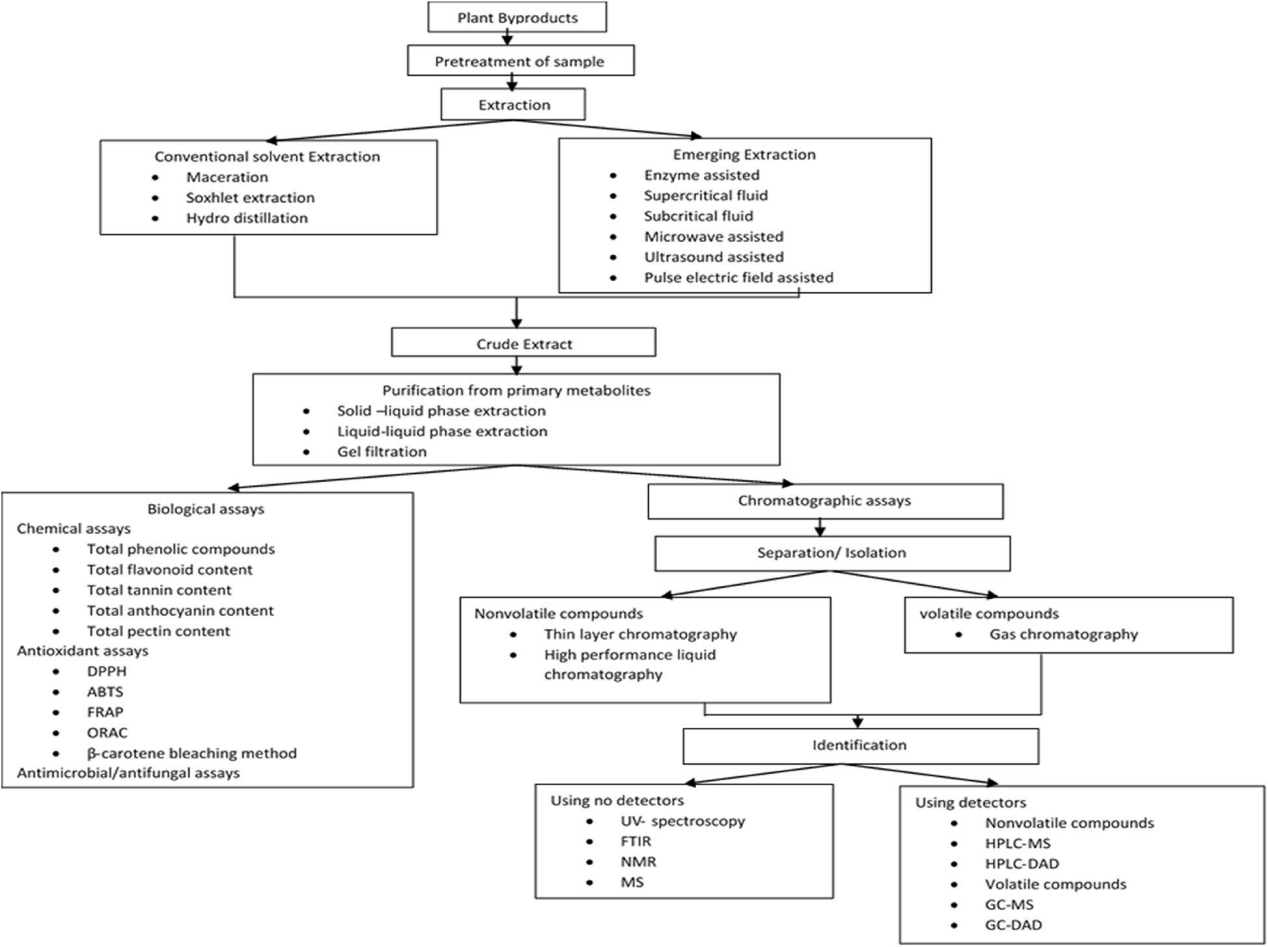
Flow chart showing the extraction and characterization of bioactive compounds from fruit by-products

The extraction technologies are categorized according to their extraction efficiency, cost-effectivity, and sustainability. Several extraction processes are followed for the recovery of bioactive compounds from the fruit industry by-products. These compounds can be separated, identified, and characterized to be utilized by different food, pharmaceutical, cosmetic or textile industries (Altemimi et al. 2017a; Marić et al. 2018). The bioactive compounds cause a lower risk of cancer, cataract, Alzheimer's, Parkinson's disease, ageing disorder, and heart-related diseases. Due to their high antioxidant activity and antimicrobial activity, these compounds perform defensive action toward chronic diseases, preventing the production of cancerous chemicals and balancing the immunosystem. These compounds are beneficial by being used as an additive in functional foods or consumed as a dietary supplement. Besides nutraceutical properties, natural antioxidants and colour compounds can be a better replacement for synthetic antioxidants, which could be used in different pharmaceutical and processing industries (Altemimi et al. 2017a; Azmir et al. 2013; Sasidharan et al. 2011). The processes starting from extraction to separation, isolation, identification, and characterization of fruit byproducts were summarized in the flow chart shown in Fig. 1. Thus, the extraction and valorization of bioactive compounds from fruit industry by-products improve societal health by providing nutritious food, mitigating the environmental problem, and reducing the waste disposal burden. It, therefore, helps the industries from an environmental and economic point of view (De Ancos et al. 2015; Kowalska et al. 2017; Trigo et al. 2020).

This review aims to provide an overview of the fruit industry by-products, a rich source of bioactive compounds. In addition, conventional (soxhlet, maceration, and hydrodistillation) and emerging (supercritical fluid, subcritical fluid, microwave-assisted, ultrasonic-assisted, enzyme-assisted, and pulsed electric field-assisted) techniques, along with separation, isolation, and identification by different analytical methods and quantification using various chromatographic and spectrophotometric methods. Furthermore, this review enlights on the mechanisms, advantages, and disadvantages of the above extraction techniques.

## By-products from fruit industries

Major industrial by-products from fruits and their nutritional significance are discussed here. Guava seeds contain around 16% oil, making them good sources of edible oil. These also contain about 61.4% crude fiber and 7.6% protein. However, until recently, guava seed oil has only been exploited to prepare mono-acylglycerol and di-acylglycerol (Raihana et al. 2015). Similarly, pomegranate seeds are a good source of high-quality oil (12–24%), polyunsaturated fatty acids (especially the conjugated punicic acid), protein (10–20%), and insoluble fibers (in which around 30–50% is cellulose and hemicellulose) (Aruna et al. 2016). Likewise, the apple seeds are rich in oil (17–23%) containing unsaturated fatty acids in high concentration and phenolic compounds, such as phloridzin and quercetin-3-galactoside fibers (20%) (Walia et al. 2014).

The by-products produced after the processing of avocados are about 21% of the weight of its fruit. These by-products are rich in carbohydrates (43–85%), lipids (2–4%), proteins (3–9%), and minerals (2–4%). In addition, the avocado peels contain a high amount of carbohydrates (44–84%), lipids (2–6%), protein (3–8%), and minerals (2–6%) (Bressani et al. 2009). On the other hand, the avocado seed kernels have 3–4% lipids, 3% proteins, 20–23% insoluble fibre, and 63–65% carbohydrates (Araújo et al. 2020). Similarly, mango's by-products constitute about 12–15% peel and 15–20% seed kernel which contains fatty acid, triacylglycerols, gallotanins, xanthone, and flavonoids (Jahurul et al. 2015). Mango peels are good sources of antioxidants, protein, pectin, which has different food and pharmaceutical applications (de Lourdes García-Magaña et al. 2013).

The papaya fruit contains 15–20% seed which is rich in oils (30%) with palmitic, stearic, oleic, linoleic acids, carpaine, glucotropacolin, benzyl isothiocyanate (BITC), caricin (sinigrin), and anzymemyosin as primary fatty acids (Vij and Prashar 2015). Pineapple peel with 30–42% fruit weight contains a high amount of cellulose, hemicelluloses, lignin, and pectin. Rambutan fruit peels contain a high amount of pectin (Maran and Priya 2014). Pear peel contains colour pigments with commercial applications. By-products of peach contain bioactive compounds, such as vitamin C and polyphenols, which show antioxidant activities (Redondo et al. 2017). Dragon fruit peels contain beta-carotene, lycopene, vitamin E, essential fatty acid and is also a good pectin source (Thirugnanasambandham et al. 2014).

## Extraction of bioactive compounds from fruit by-products

**Fig. 2:**
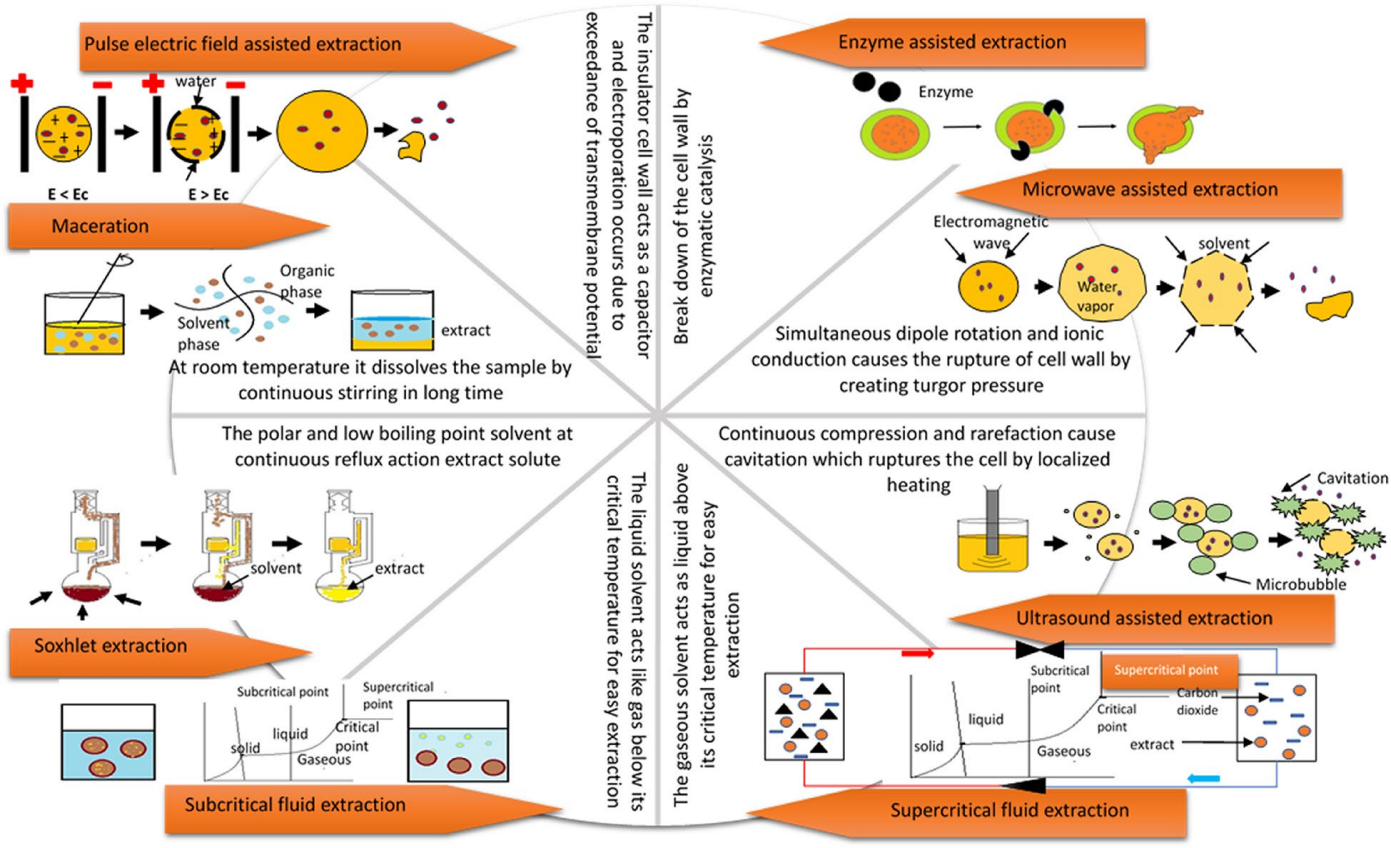
Mechanism of conventional and emerging extraction technologies with schematic diagrams

Different extraction technologies are utilized to extract beneficial compounds, especially bio-actives, present in the innermost portion of the cell of fruit by-products. A schematic diagram of available extraction technologies along with their mechanism of action is given in Fig. 2. The selection of technology for the extraction process is based on the required degree of purity of the extract, physical and chemical properties of the compound of interest, the location of the compound to be extracted (i.e., either it is free or it is bounded inside the cell of the by-products), cost-effectiveness and value of the extracted product. Before extraction processes, there are several unit operations to be done for better yield. Washing, cutting, size reduction, and drying are examples of various unit operations (Gong et al. 2020).

The standard conventional extraction techniques take a lot of time, energy, and solvent during processing and have some drawbacks. Due to this, emerging technologies are popularly used these days in pharmaceuticals, food, and medicinal industries. They require fewer solvents, less extraction time, and have more extraction efficiency than conventional technologies (Belwal et al. 2020). These technologies are selected based on their advantages, disadvantages, operation principles, and equipment types available for extraction. The operation parameters, advantages, and disadvantages of different extraction technologies are described in Table 1.

**Table1:** Process parameters, advantages, and disadvantages of different extraction techniques

Parameters involved	Advantages	Disadvantages	References
*Conventional solvent extraction*			
Plant materials (moisture content, particle size, pH) Solvent parameters (solid: solvent ratio, choice of the solvent, boiling point of the solvent, mixing the composition of solvents) Process parameters (temperature, pressure)	Low cost Simple operation Suitable for thermolabile compounds Ease extraction of flavonoids Enviro-friendly because of solvent usage as water	A tremendous amount of solvent is required Higher extraction time and energy Low extraction efficiency Higher toxicity Polar solvents are commonly used Degradation of extracts Several additional steps required for extraction Solvent presence in extracts	(Ares et al. 2018; Gu et al. 2019; Mehmood et al. 2019; Taofiq et al. 2019)
*Enzyme-assisted extraction*			
Plant materials (moisture content, nutrient availability, particle size) Enzyme parameters (type, concentration, composition, characteristic properties, mixing ratio) Solvent (types of solvent, solvent ionic strength) Process parameters (temperature, pH, process time)	High recovery of phytochemicals Low energy and time consumption No requirement of additional steps Low residual levels Not altering the structure of the cell Limited equipment corrosion No toxic waste Low Temperature and Cost	Cost of enzymes is relatively higher Can't completely hydrolyze the structure of the cell wall Scale-up process High disbursement for the drying after the enzyme treatment Very long extraction time of 1–4 h Filtration must be required	(Adetunji et al. 2017; Gong et al. 2020; Grassino et al. 2020; Nadar et al. 2018; Panja 2018)
*Supercitical Fluid extraction*			
Plant materials (particle size, moisture content, surface area, porosity) Solvent parameters (density, diffusivity, viscosity, polarity, compressibility, solvent mixture ratio, flow rate, vapour pressure) Process parameters (temperature, pressure, dynamic time)	CO_2_ is chemically inert, non-toxic, cheap, well-accepted food-grade solvent Low polarity, high diffusivity solvent Allowing low polar molecules with additional clean set-up Inactivation of microorganisms Low cost, low consumption of chemicals Clean and free solvent extract Reduce the oxidation of component Can penetrate faster than the other solvent	It is a non-polar solvent so that it can't extract polar extracts Requires modifiers Complex equipment Barely applied for extraction and fractionization of carbonyl The presence of water may cause problems Risk of volatile compounds High capital investment and cost Elevated pressure is required Many parameters to optimize	(Al-Otoom et al. 2014; Gere et al. 1997; Pinto et al. 2020; Pourmortazavi and Hajimirsadeghi 2007)
*Subcritical fluid extraction*			
Plant materials (particle size, moisture content) Solvent parameters (temperature, critical pressure, mixing, flow rate, modifiers, additives) Process parameters (modes, time of extraction, Solid: liquid ratio)	Shorter extraction time Higher efficiency The continuous process is possible Less expensive instrument Polar, moderately polar solvent Low polar and non-polar compounds can be extracted separately Water is an extraction solvent that has the benefits of being green, cheap, and readily available	Equipment is not easy to clean More reactive and corrosive Thermal degradation may occur at high temperature Improper control process condition Hydrolysis of the pectin chain Not easy to remove moisture from the extracts and may require additional procedures, including evaporation, dehydration, precipitation	(Essien et al. 2020; Gallego et al. 2019; Zhang et al. 2020)
*Microwave-assisted extraction*			
*Plant materials (moisture content, particle size, texture complexity, dielectric loss factor)* *Solvent parameters (pH, volume, dielectric properties, solid: solvent ratio, density)* *Process parameters (microwave power, temperature, pressure, time of extraction)*	Heating is homogenous No temperature gradient between solid and solvent system Minimized solvent use Lower time of extraction and cost Improved extraction yield Simple in operation Minimum labour used No risk of oxidation Volumetric heating Volatile compounds can be removed without any loss	Solvents solution may cause wear in types of equipment Not suitable for thermosensitive compounds Sometimes fat oxidation occurs To collect the volatile compound, the vessel is to be cooled Solution temperature is low	(Al-Dhabi and Ponmurugan 2020; Arrutia et al. 2020; Okolie et al. 2019; Upadhyay et al. 2012)
*Ultrasound-assisted extraction*			
Plant materials (emulsifying capacity, solubility texture, liquid: solid ratio, presence of dissolved gases) Solvent parameters (type of solvent, mixture ratio, reaction of solvent towards the target compounds) Process parameters (frequency range, frequency combination, vibration of the probe, amplitude displacement, ultrasonic generator, ultrasound intensity, shape and size of the ultrasonic reactor, pressure, temperature, pump flow)	Improve the efficiency Reduce time of extraction and cost Increase the extraction yield Reduce the amount of solvent Versatile in terms of solvents Rapid and reproducible Prevention of material and solvent wastage Lower process temperature No costly equipment Lower environment polluting risks Less expensive than microwave digester Replaceable with GRAS solvents Used for thermolabile compounds	The repetition of the process would be done Non-uniformity of ultrasound with solution Harmful for human health by toxic solvent More solvent will be required Filtration is needed The target compounds are sometimes overcooked Generation of oxidizing species (OH^+^, H_2_O_2_) for the oxidation of organic material	(Belwal et al. 2020; Carrillo-Hormaza et al. 2020; Chakraborty et al. 2020; Dzah et al. 2020; Meregalli et al. 2020; Saifullah et al. 2020)
*Pulsed electric field assisted extraction*			
Plant materials (the moisture content of biomass, Size, and shape of the plant cell) Solvent parameters (solvent type, solvent: sample ratio) Process Parameters (flow rate, frequency, extraction time, temperature, the diameter of the chamber, numbers, and duration of electric pulses)	Cheaper and usage of less energy Non-toxic Environment-friendly solvents usage Moderate temperature Improving productivity Higher selectivity for intracellular products Increase the extraction rate Decrease the amount of solvent Shortening the extraction time Avoiding quality loss and fractionization of non-pure extracts	The high cost of equipment A high voltage pulse is required Insulation should be there Electrode usage causes corrosion Dependence of medium Composition or conductivity of solvent	(Andreou et al. 2020; El Kantar et al. 2018a; Medina-Meza and Barbosa-Cánovas 2015; Plazzotta et al. 2021)

Mainly the extraction process has the following objectives: (a) to extract the targeted bioactive compounds from complex plant samples, (b) to increase the selectivity of analytical methods, (c) to increase the sensitivity of bioassay by increasing the concentration of targeted compounds, (d) to convert the bioactive compounds into a more suitable form for detection and separation, and (e) to provide a reproducible and robust method that is independent of variations in the sample matrix (Azmir et al. 2013). However, the basic mechanisms of all the solvent extraction processes are: (1) the solvent penetrates the solid matrix; (2) the compound of interest dissolves in the solvents; (3) the compounds diffused out of the solid matrix; (4) the extracted compounds are collected (Smith 2003).

The factors affecting the different extraction process is illustrated in Table 1. Besides those, the primary factors influencing extraction efficiency are the type of solvents used, extraction time, the particle size of the sample, temperature of extraction, and solid/solvent ratio (Garavand et al. 2019). The particle size must be small to penetrate the solvent inside the sample, and the temperature should be high for a higher yield. But too much high temperature can cause the loss of volatile compounds during the extraction process. Too much duration cannot affect the extraction, since the extraction process attains an equilibrium state after a specific time. The ratio between solid and solvent should be moderate; a too-high ratio might take more time for extraction (Zwingelstein et al. 2020).

Industrial scaling up of the extraction requires extensive consideration of both economy and productivity, while the lab-scale extractions require only a small amount of raw materials and solvent. Several factors are considered during the large-scale extraction process, such as instrumentation, type of process (batch or continuous), kinetics, economics, and energy consumption (Belwal et al. 2020; Chemat et al. 2012). There have been many investigations about scaling up the extraction processes. About 60% of industrial applications are done with SFE, while 15% and 14% of extraction are carried out with ultrasound and microwave, respectively (Belwal et al. 2020; Chemat et al. 2019). In the case of the UAE, both probe and bath systems are used on pilot / industrial scales, and microwave reactors are usually preferred in industrial proposed extraction. Meanwhile, UAE is the primarily used extraction process for industrial-based juice production. However, in the case of PEF-assisted extraction process, there are still challenges for the industrial scaling up of the process.

## Bioactive compounds present in fruit by-products

The bioactivity of fruits and vegetables is defined as the capacity to counteract the adverse effects of oxidative stress on human health, such as several human diseases, such as diabetes, cancer, cardiovascular diseases, osteoporosis, etc. The bioactive compounds are a secondary metabolite of the plant and can be classified into essential and non-essential nutrients. The phenolic compounds, dietary fibers, and fatty acids are non-essential, while the vitamins and minerals are essential nutrients (Guil‐Guerrero et al. 2016; Padayachee et al. 2017).

The byproducts of fruits contain phytochemicals comprising phenolic compounds, vitamins, minerals, dietary fiber, and other bioactive compounds. The polyphenolic compounds present inside the fruit cause immunity towards the development of human health. The phenolic compounds are the secondary metabolite of the fruits, which can act against the free radicals and oxidative stresses, and hence they are called antioxidants (Singh et al. 2017; Trigo et al. 2020). These are structurally complex compounds containing high molecular weight phenols which bear at least one or more hydroxyl groups in the aromatic ring. Commonly, the phenolic compounds are in the form of flavonoids, phenolic acids, and tannin (Babbar and Oberoi 2014). The phenolic acid is formed by one phenolic ring with a carboxylic acid. Some examples of phenolic acids are hydroxybenzoic acid, hydroxycinnamic acid, gallic acid, ferulic acid, and cinnamic acid. These compounds exhibit higher antioxidant and antimicrobial activity, which mainly depend on the position of the hydroxyl and carboxyl groups and the double bonds present in the ring (Akhtar et al. 2015; Sanchez-Maldonado 2014). Besides phenolic acids, flavonoids also have biological properties, such as antioxidative, antimicrobial, anticancerous, and cardioprotective. Flavonoids are heterocyclic aromatic rings comprising flavonol, flavanone, flavones groups, where catechin, hesperetin, and proanthocyanidin are a few examples (Yalcin and Çapar 2017). Tannins are water-soluble phenolic compounds divided into galotannins, ellagitannins, condensed tannins, and complex tannins. These compounds also exhibit antioxidant activity due to the presence of the phenol hydroxyl groups, which are capable of reducing the free radicals and antimicrobial activity by inactivating the enzymes as well as precipitating the proteins (Akhtar et al. 2015; Guil‐Guerrero et al. 2016).

Anthocyanins are water-soluble plant pigments of red, purple, and blue colour, derived from the peel of fruits, such as pear, watermelon, and apple. Similarly, carotenoids are another water-insoluble/ Lipid soluble plant antioxidants that are the precursors of vitamin A. Carotene (lycopene, β-carotene, α-carotene), xanthophyll (lutein) are some examples of carotenoids present in seeds and peels of the fruit. The arrangement of conjugated double bonds is responsible for the antioxidant activity of the carotenoids (De Ancos et al. 2015). Vitamins present in fruits and vegetables are responsible for preventing lipid oxidation, decreasing DNA damage, and maintaining immune function. Ascorbic acid and tocopherols are precursors of vitamin C and vitamin E, respectively. α, β, γ, δ tocopherols are four analogues that are responsible for hypolipidemic, antiatherogenic, antihypertensive, allergic dermatitis suppressive, neuroprotective, and anti-inflammatory activities (Golkar and Moattar 2019). The polysaccharides present in the cells of fruit by-products are responsible for the antihyperlipidemic, prebiotic, antitumor activities, and jellification and emulsification efficiencies. The polysaccharides are classified into water-soluble (pectin) and water-insoluble (cellulose, lignin) groups. Pectin is a heterogeneous type of acidic polysaccharides present in the cell lamella of fruits. Besides these, the oils extracted from the seed or peel powder, described in Table 2, have antioxidant, anticancerous, and antidiabetic properties because of the presence of fatty acids, such as polyunsaturated (linoleic acid, linolenic acid), and monounsaturated (oleic acid) (Golkar and Moattar 2019; Minh et al. 2019; Peso-Echarri et al. 2015; Yalcin and Çapar 2017).

**Table 2:** List of separation, identification, characterization, and quantification of extracted bioactive compounds from fruit byproducts (seed, peel)

Sl. No.	Name of the fruit and its by-products	Separation and Identification	Characterization of bioactive compounds	Major bioactive compounds	References
*Conventional extraction*					
1	*Citrus sinensis* (Orange*),* *Citrus* limonia (lemon*)* *Musa acuminate* (banana) peel	GC- MS analysis Atomic Absorption spectroscopy	Antimicrobial activity (MIC-130 µg/mL) (*Klebsiella pneumoniae*), Total phenolic content (29.72 mg GAE/g)	Coumarine Tetrazene Mineral (14.5 (Zn) ppm, 19.32 (Mg) ppm)	(Mehmood et al. 2019)
2	*Psidium guajava* (Guava) seed	HPLC	Total phenolic compounds (Folin-Coicalteu method) (124.03 mg GAE/100 g) Antioxidant activity (DPPH method) (58.90%) Acid value ( 0.40 mg KOH/mg) Peroxide value ( 0.62%)	Linoleic acid ( 75.25%) Oleic acid (11.40%) Palmitic acid (6.6%) 2-Chloroethyl linoleate (44.53%), 2-Pentanone,4-hydroxy-4-methyl (23.56%) n-Hexadecanoic acid (9.85%) Tocopherol (α-tocopherol) (654 ppm) Caretonoids (β-carotene) (19.24 ppm) Phytosterol (stigmasterol, campesterol) (420 mg/100 g)	(Kapoor et al. 2020)
3	*Punica granatum* (Pomegranate) seed		Edible oil with high nutrition acid value (0.83 mg KOH/g) iodine value (107.18 mg/100 g) peroxide value ( 3.84 meq O_2_/Kg) Saponification value (189.17 mgKOH/g)	Oleic acid ( 48.54%) Linoleic acid ( 23.40%) Palmitic acid ( 15.47%) Stearic acid ( 8.14%)	(Lucci et al. 2015)
4	*Myrciaria dubia* (Camu-camu) seed	HPLC–DAD	Antiparasitic activity (spectrophotometric method) Antioxidant activity (DPPH) (77%) Total phenolic compounds (5619 mg GAE/100 g) Total flavonoid content (1700 mg CE/100 g) Total tannin content (1328 mg CE/100 g)	Vescalagin,Castalagin Gallic, ellagic acids, quercetin-3-rutinoside, malvidin-3,5-diglucoside and cyanidin-3-O-glucoside	(Fidelis et al. 2020)
5	*Passiflora edulis f.Flavicarpa L.* (Passion) peel		Pectin production (calcium pectate) Methoxyl content (9.6 g/100 g) jelly grade equivalents (200) equivalent of galacturonic acid (88.2 g/100 g)	Pectin ( 14.8 g/100 g)	(Kulkarni and Vijayanand 2010)
6	*Annona squamosa* (Pomegranate) peel		Antioxidant activity (DPPH) (24.54%) Total phenolic compounds (510 mg GAE/g) colour index (ΔE) (4.07) Flavonoid content (16.40 mg quercetin/gm)	Phenolic compounds Flavonoids Reducing sugar (0.18 mg of inert sugar)	(Sood and Gupta 2015)
*Enzyme-assisted extraction*					
7	*Phoenix dactylifera* (Date) seed		Bio-based fuel (saccharification efficiency) (31 g/mL)	lignin 27.34% cellulose 20.63% hemicellulose 13.49%	(Hamid and Ismail 2020)
8	*Passiflora edulis f* *Flavicarpa* (Passion) peel		Pectin production (calcium pectate) Galacturonic acid (85 g/100 g) Degree of esterification (68%)	Pectin (26 g/100 g)	(Vasco-Correa and Zapata 2017)
9	*Punica granatum* (Pomegranate) seed	MS	Antioxidant activity (DPPH) (98.3%) Total phenolic compound (432 mgGAE/g)	Linoleic acid Linolenic acid Punicic acid Leucine Phenylalanine Glutamic acid	(Talekar et al. 2018)
10	*Vitis rotundifolia* (Grape) peel	ESI- MS	Antioxidant activity (DPPH), Total phenolic compound anthocyanin content	Phenolic compounds Anthocyanin	(Xu et al. 2014)
11	*Punica granatum* (Pomegranate) peel	HPLC–DAD-ESI–MS	Antioxidant activity (DPPH, ABTS) acid, (445.02 mol TE/g) (IC_50_ 50.15 ± 0.01 µg/mL) Total phenolic compounds (301.53 mgGAE/g)	Vanillic acid (108.36 µg/g) Synergic acid (75.19 µg/g) Ferulic acid (88.24 µg/g)	(Mushtaq et al. 2015)
12	*Anana scomosus* (Pineapple) peel	HPLC–MS	Vinegar production sugar yield (70.2 g/kg) saccharification efficiency	D-fructose D-glucose	(Roda et al. 2016)
*Supercritical fluid extraction*					
13	*Butia catarinensis* (Jelly palm) seed	GC–MS	Antioxidant activity (195.6 µM TEAC/g) Antimicrobial activity (agar diffusion, MIC) () Total phenolic compounds (22.6 mg GAE/g)	Camphene Caprylic acids Epicatechin Gallic acid P-hydroxybenzoic acid	(Cruz et al. 2017)
14	*Psidium guava* (Guava) seed	RP-UHPLC-DAD-HESI-MS/MS, RP-UHPLC-HESI-IT-FTMS	Total phenolic compounds (263.1 mg GAE/100 g) Antioxidant activity (58.90%)	Linoleic acid (78.5%) Oleic acid (13.8%) Vanillin (9.6 mg/100 g) Vanillic acid (3.9 mg/100 g) Cinnamic acid (2.4 mg/100 g) Cinnamaladehyde (9.4 mg/100 g) β-sitisterol (1048.9 mg/100 g) γ-tocopherol (82.6 mg/100 g)	(Narváez-Cuenca et al. 2020)
15	*Cucurbita maxima* (Pumpkin) peel	GC–MS	antioxidant activity (DPPH) (IC_50_ 788.8 µg/ml) Total phenolic compounds (1053.18 mg GAE/100 g)	Oleic acid (27.1%) Linoleic acid (13.4%) Linolenic acid (4.5%) β-carotene (375.8 mg/100 g) γ-tocopherol (280.3 mg/100 g) Campesterol (16.9 mg/100 g) Stigmasterol (250.6 mg/100 g) Palmitic Acid (32%) Stearic Acid (0.3%) Arachidic Acid (0.1%)	(Cuco et al. 2019)
16	*Malus pumila* (Apple) seed	GC, HPLC–MS	Antioxidant activity ( DPPH (0.71 µg TROLOX/g)), FRAP (0.63 µg TROLOX/g)) Total phenolic acid (1.61 µg GAE/ g), oil stability (on set time 21.4 h)	Linolenic acid (63.76 g/100 g) Phloridzin (2.96 µg/g) Amygdaline Oleic acid (34.84 g/100 g)	(Ferrentino et al. 2020)
17	*Punica granatum* (Pomegranate) seed	GC	Antioxidant acivity (DPPH) Oxidant stability (3.5 ± 0.6 mg α_-toc_/mLoil)	Palmitic acid (3.3%) Stearic acid (1.49%) Oleic acid (3.9%) Linoleic acid (5.9%) Punicic acid (85.4%)	(Natolino and Da Porto 2019)
18	*Fragaria ananassa* (Strawberry) seed	HPLC	Total phenolic acid (1429 mg GAE/100 g) Flavanol (5.82 g/kg DW)	Ellagitannins (8.50 g/kg DW) Anthocyanins (152 mg/kg DW) Kaempferol-3-O-b-D- (6’’-E-p-coumaroyl)-glucopyranoside (184.3 mg/100 g)	(Grzelak-Błaszczyk et al. 2017)
19	*Rubus idaeus L* (Raspberry) seed	HPLC-FLD, GC	Antioxidant activity (DPPH) ( 9.12 µM/g) tocopherol content ( 166.34 mg/100 g)	ώ-3 fatty acid (18.64%) Linoleic acid ( 55.49%) Linolenic acid ( 34.76%) Palmitic acid ( 3.55%) Stearic acid ( 1.29%) Oleic acid ( 13.67%) γ-tocopherol ( 110.42%)	(Marić et al. 2020)
20	*Vaccinium myrtillus* L (Bilberry) seed	HPLC	Antioxidant activity (DPPH) (IC_50_ 9.5 mg/mL), Fatty acid stability tocopherol content (70 mg/100 g)	Linoleic acid (36.2%) α-Linolenic acid (23.2%) Oleic acid (33.6%) γ-tocopherol (56.8 mg/100 g) Lutein β-cryptoxanthin β-carotene	(Gustinelli et al. 2018)
21	*Eugenia uniflora* L (Brazilian cherry) seed	TLC, GC–MS	Antioxidant activity (78%) refractive index (1.519)	γ-element (0.0091 mglimEq/100 g) Germacrone (0.0088 mg limEq/100 g)	(e Santos et al. 2015)
*Subcritical fluid extraction*					
22	*Carica papaya* (Papaya) seed	LC–ESI–MS/MS	Total phenolic compound (47.7 mg GAE/g) Antioxidant activity (DPPH (2.9 µg/ml), β-carotene bleaching assay (98%) milliard reaction	Ferulic Acid (0.1087 mg/g) Mandelic Acid (0.1227 mg/g) Vanillic Acid (0.1082 mg/g)	(Rodrigues et al. 2019)
24	*Passiflora alata Curtis* (Sweet passion) seed	TGA, GC–MS	Antioxidant activity (DPPH (78.78%), ABTS (172 µM TEAC/g), tocopherol content (11.14 mg/100 g oil) fatty acid stability (1.51 mg KOH/g oil)	δ-Tocopherol (6.85 mg/100 g oil) Linoleic acid (72.04%)	(Pereira et al. 2017)
25	*Psidium guava* (Guava) seed & peel		Total phenolic compounds (124 mgGAE/g)	Tocopherol	(Duba et al. 2015)
26	*Citrus grandis* (Pomelo) peel	HPSEC-MALLS	Pectin production (19.6%) degree of esterification (38.2%) Galacturonic acid (76.62%)	Isomethyl pectin	(Liew et al. 2018)
27	*Citrus Junos* (yuzu) peel	ATR-FTIR, TG–DTA	Pectin production (78%)	Cellulose (80%) Hemicelluloses	(Tanaka et al. 2012)
*Microwave-assisted extraction*					
28	*Syzygium cumini* L (Jamun) seed	FTIR	Polysaccharide production (4.72%)	Polysaccharide	(Al-Dhabi and Ponmurugan 2020)
29	*Mangifera indica* (Mango)seed	RP-HPLC–ESI–MS	Antioxidant activity (ABTS 1738.2 mg trolox/g) IC_50_ (0.078 mg/mL)	Ethyl gallate Penta-*O*-galloyl-glucoside Rhamnetin-3-[6’’-2-butenoil-hexoside]	(Torres-León et al. 2017)
30	*Hylocereus spp.* (Dragon fruit) peel	FTIR	Pectin production (23.11%) degree of esterification (73%) Anhydrunic acid (62%)	Methoxyl pectin	(Tongkham et al. 2017)
31	*Persea americana Mill* (Avocado) seed	HPLC–ESI–MS	Phenolic content (307 mg GAE/g) Antioxidant activity (DPPH (226 mg ET/g), ABTS (607 mg ET/g), ORAC (495.25 mg ET/g))	Procyanidins, Catechin, Epicatechin, Perseitol, Hydroxy benzoic, Hydroxycinnamic acids	(Araújo et al. 2020)
32	*Citrullus lanatus* (Watermelon) peel		Pectin production (25.79%)	Methoxyl pectin	(Maran et al. 2014)
33	*Anana scomosus* (Pineapple) peel		Phenolic compounds (113.65 mg GAE/g) Antioxidant acivity (DPPH) (62.37 µM trolox/g) Methoxyl pectin (6.12%) Galacturonic acid content (44.78%) Degree of esterification (39.39%)	Pectin	(Rodsamran and Sothornvit 2019b)
34	*Phaleria macrocarpa* (Mahkota dewa fruit) peel		Total phenolic compounds (102.60 mg GAE/g), Antioxidant activity (61.15% DPPH scavenging activity)	Phenolic compounds	(Alara et al. 2019)
*Ultrasound-assisted extraction*					
35	*Nephelium lappaceum L*. (Rambutan fruit) peel	FTIR	Polysaccharide production (8.29%)	Polysaccharide	(Maran and Priya 2014)
36	*Ziziphus jujube* (Jujube) seed	Ion-exchange and gel-permeation chromatography, FTIR, HPLC, NMR	Polysaccahride production (1.95%) Antitumor activity (MIC 63.37%) Antioxidant activity (IC_50_ 164.6 µg/mL)	Arabinose (21.63 mmol/L) Glucose (9.81 mmol/L) Xylose (13.52 mmol/L) Galactose (15.28 mmol/L) Rhamnose (8.73 mmol/L)	(Wu et al. 2019)
37	*Syzygium cumini* (Jamun) seed		Phenolic compounds (100.07 mg GAE/g), Antioxidant activity (DPPH) (IC_50_ 10.59 µg/g) Flavonoids (AlCl_3_ method) (10.11 mg catechin/g),	Gallic acid (54.5 mg/g) Ellagic acid (2.2 mg/g)	(Mahindrakar and Rathod 2020)
38	*Adansonia digitate* (Baobab) seed	HPLC	Antioxidant activity (DPPH 322.12 mg Trolox/100 g, FRAP 1659.72 mg Trolox/100 g) Flavonoid content (1633.84 RE/100 g) Total phenolic compound (418.01 mgGAE/100 g)	D-catechin (87.18 µg/g) Chlorogenic acid (4.67 µg/g) Caffeic acid (3.12 µg/g) Felluric acid (2.55 µg/g) Rutin (0.82 µg/g) procatechuic acid (10.63 µg/g)	(Ismail et al. 2019)
39	*Hylocereus undatus* (Dragon fruit) peel		Antioxidant Activity (DPPH, FRAP), Phenolic compounds	Phenolic compound	(Raj and Dash 2020)
40	*Mangifera indica* (Mango) peel		Antioxidant activity (DPPH (3.18 mg TROLOX/100 g)) Total phenolic compounds (1493.01 mg GAE/100 g)	Phenolic compounds	(Martínez-Ramos et al. 2020)
41	*Pouteria campechiana* (Canister) peel	HPLC, FTIR, microscopy, NMR spectroscopy	Polysaccharide production (15.94%) Antioxidant activity IC_50_ (DPPH (10.31 µg/ml), ABTS (29.9 µg/mL))	Glucose Mannose	(Ma et al. 2020)
42	*Psidium cattleianum* *Sabine* (Strawberry guava) peel		Antioxidant activity (DPPH) (86.31%) Total phenolic compounds (589.49 mg GAE/100 g) Flavonoids (374.05 mg de catechin/ 100 g of peel) Caretonoids (4.47 mg of b-carotene/ mL)	Anthocyanin ( 121.85 Eq. mg of cyanidin-3-glycoside/100 g) Catechin β-carotene	(Meregalli et al. 2020)
*Pulsed electric field-assisted extraction*					
43	*Malus domestica* (Apple) peel		Antioxidant activity confocal laser scanning microscopy,	Solid concentration Phenolic compounds	(Wang et al. 2020)
44	*Carica papaya* (Papaya) peel		Antioxidant activity (DPPH) (78%) Total phenolic compounds (15.18 mg GAE/l)	Isothiocyanate (42.67 µmol/100 g) Protein Carbohydrate	(Parniakov et al. 2015)
45	*Opuntia stricta* (Pear) peel	HPLC	Electro scanning microscopy	Red colourants (betanin, isobetanin) (81.3 mg colourant/100 g)	(Koubaa et al. 2016)
46	*Mangifera indica* (Mango) peel		Protein (Bradford method) (280 mg/kg), Phenolic (Folin-coicalteu method) (2001 mg GAE/kg), and polysaccharide (phenol–sulphuric method) (204 g/kg), Antioxidant activity (DPPH) (9.5 mM TE/g)	Pectin Phenolic compounds	(Parniakov et al. 2016)
47	*Citrus sinensis* (Orange) peel	HPLC–DAD	Phenolic compounds (23 mg GAE/100 g) Antioxidant activity (DPPH) (2.34 mg TROLOX/100 g)	Naringenin (3.6 mg/100 g) Hesperin (4.8 mg/100 g)	(Luengo et al. 2013)

## Characterization of bioactive compounds

After extraction, the bioactive compounds are separated, purified, and identified to determine the presence of specific compounds in specific quantities. These compounds are also categorized by functional activities, which are determined by different bioactivity assays. Many analytical methods are used for the separation, purification, and identification of bioactive compounds, but the screening of those methods are done according to the simplicity, specificity, and speed (Altemimi et al. 2017a; Sasidharan et al. 2011). Column chromatography techniques are used to separate and isolate the desirable compounds from the mixture of extracts. The bioactive compounds are separated and purified based on their adsorption properties, molecular size, ionic strength, boiling points, and so on (Zhang et al. 2018). Adsorption column chromatography is the technology in which the target molecules are separated based on affinities towards the adsorbent surfaces, such as silica gel, aluminium oxide, polyamides, and silver nitrates. On the other hand, the partition chromatography and counter-current chromatography are based on the liquid–liquid extraction in which one liquid phase is stationary, and another liquid phase is in the mobile phase. Moreover, in the case of membrane filtration and gel chromatography, the bioactive compounds are separated by molecular size. The smaller molecule can pass through while the large molecular particles are retained. In the case of gel chromatography, the bioactive compounds are purified according to their retention time. Meanwhile, the ion-exchange chromatography separates the bioactive compounds based on net surface charge. In this, the molecules can be caught or released by ion-exchange resin by changing the ionic strength of the mobile phase. However, in the case of distillation, the thermosensitive or high molecular weight bioactive compounds can be separated (Altemimi et al. 2017b; Mahato et al. 2019; Raje et al. 2019). For identification and quantification, the bioactive compounds are determined using several spectroscopic techniques. There are various bioactivity assays for measuring its functional activities, such as antioxidant activity, antimicrobial activity, and so on. Before all the above procedures, the extracts are first refined from interfering common metabolites using different pretreatment procedures to make the preconcentration of secondary metabolites. Moreover, the quantification of these compounds is also demonstrated by many chemical assays (Bailey 2015; Roberts and Caserio 1977).

The chemical assays are demonstrated for the determination of phenolic compounds, tannins, vitamins flavonoids, pectin, and fatty acid content in various analyses in which the quantities of those compounds are calculated using the equivalent of standards, such as gallic acid, tannic acid, catechin, galacturonic acid, oleic acid and so on. After that, there are several bioactivity assays, such as different radical scavenging (DPPH, ABTS), oxygen reducing power (ORAC), carotene bleaching, iron-chelating (FRAP) assays, microbial inhibition capacity for determination of antioxidant activity, antimicrobial activity, respectively (Ivanović et al. 2020; Trigo et al. 2020).

The separation and isolation of bioactive compounds are determined by chromatographic methods, distinguished by their polarity. Gas–liquid chromatography (Gas chromatography) is used, while the extract contains some slight volatile compounds and liquid–solid chromatography [thin-layer chromatography (TLC), high-performance liquid chromatography (HPLC] are used, while the mixture contains high molecular weight molecules (Altemimi et al. 2017a). The presence of polar compounds in those compounds is separated to the other side of the column, leaving the mixture.

Finally, after the isolation of bioactive compounds, various spectroscopic methods are used for identification, composition, and bonding inside the molecules. The basic principle of these methods is the absorption of electromagnetic radiation by the molecules, which gives a spectrum. The spectra are meant for identification as every spectrum is specified for each type of bonding in the molecule. UV–visible, Fourier transform Infrared spectroscopy (FTIR), Nuclear Magnetic Resonance (NMR), and mass spectroscopy (MS) are some examples of those methods (Ivanović et al. 2020; Sasidharan et al. 2011).

However, the combination of sensitive and rapid analytical techniques with spectroscopic methods is popularly used for rapid identification and quality control of complex extract. HPLC/GC is widely used for isolation, identification for quantitative and qualitative analysis of extract; it is coupled with MS, FTIR, NMR, and so on (Trigo et al. 2020). To increase the speed of analysis, higher separation efficiency, and sensitivity, ultrahigh-pressure liquid chromatography is achieved. In addition, for detection of chromophores (polyphenols, flavonoids, alkaloids, quinones) and non-chromophores (terpenes, saponins), the detectors, such as UV-diode ray (DAD), Evaporative Light Scattering Detector (ELSD), Electron Capture Detector (ECD), MS, NMR and so on. The selection of detectors depends on nature, properties of bioactive compounds, their sensitivity, and the proposed information to be needed (structure, quantification) (Altemimi et al. 2017a; Ivanović et al. 2020; Sasidharan et al. 2011). The separation, identification, characterization of bioactive compounds from fruit byproducts (seed, peel) using several extraction methods are described in Table 2.

## Effect of extraction technologies on bioactive compounds

### Conventional extraction (CE)

Different CE processes used to separate bioactive compounds from plant materials are maceration, soxhlet extraction, percolation, infusion, digestion, decoction, and heat reflux extraction (Mehmood et al. 2019). These extraction processes are also called solvent extraction processes, because they require more amounts of mild or more polar solvents for extracting directly from biomass. Different types of solvents used in these processes are ethanol, methanol, hexane, n-butane, petroleum ether, and water. Besides the above conventional extraction processes, some processes where water is used as solvent are hydro distillation, steam distillation, steam water distillation, and steam diffusion. Different processes are distinguished by the amount of solvent used, types of equipment used, and compounds extracted. Soxhlet extraction is more suitable than maceration due to less solvent used, but it can degrade some amount of natural compounds due to high temperature. Hydrodistillation is used to separate volatile organic compounds, since it requires only less time for extraction. Maceration is used to remove thermolabile compounds, since it requires only normal room temperature (Memarzadeh et al. 2020; Taofiq et al. 2019).

The main factors that affect the CE processes are the type of solvent and its characteristics (Gu et al. 2019; Zengin et al. 2020). Lower polar solvents (petroleum ether, chloroform, etc.) extract lipophilic compounds and certain pigments, such as carotenoids and chlorophyll. However, high polar solvents are generally used to extract bioactive compounds (Ares et al. 2018). Moreover, they are best for extracting flavonoids and anthocyanins due to their acidified condition (Okolie et al. 2019).

Fruits such as orange, lemon, and banana are highly nutritious and provide health benefits to humans. The peels of these fruits are composed of phenolic compounds and minerals which show antimicrobial properties and are beneficial for therapeutic purposes (Parashar et al. 2014). Saleem and Saeed (2020) conducted a study on extracting bioactive compounds from fruit peels using CE technologies with methanol, ethyl acetate, ethanol, and distilled water. They reported that the extracted compounds exhibited antimicrobial properties. The microbial inhibition concentration (MIC) was recorded as 130 µg/ml during testing the microorganism (*Klebsiella pneumoniae*). At the same time, the water was considered a solvent in the extraction of yellow lemon peels. For the extraction, the solvent used were methanol, ethyl acetate, ethanol, and distilled water.

Similarly, another study reveals that the guava seed, a by-product of the guava juice industry, has an oil content of 16%. In addition, the yield of oil extracted was higher (13.63%) during soxhlet extraction than supercritical fluid extraction. Furthermore, the oil was extracted using n-hexane for 60 °C up time 4 h in a soxhlet extractor (Kapoor et al. 2020).

Lucci et al. (2015) extracted oil from pomegranate seed from solvent extraction, taking ethanol as a solvent containing bioactive lipid compounds, such as punicic acid, glycolipid–linoleic, a-linoleic acids phospholipids. They also found that the extracts show antioxidant and antiproliferative activities against human cancer cells. Another study reported that the passion fruit peels are rich in high methoxyl pectin, which was extracted at pH 2.0, peel to solvent ratio of 1:30 (w/v), extraction temperature of 98.7 °C and time of extraction of 60 min, where the solvent was taken as acidified water with hydrochloric acid (Kulkarni and Vijayanand 2010). Sood and Gupta (2015) used the ethanolic solution to extract bioactive compounds from pomegranate peel. They reported that the extracted compound is a good source of phenolic and flavonoids content (quercetin) which was opted at the conditions of solid to solvent ratio of 1:30, the temperature of 50 °C, and the time of extraction of 45 min.

In the same way, Fidelis et al. (2020) utilized the solvent extraction method, with the combination of three solvents, i.e., ethanol, water, and propane, to extract the bioactive compounds from camu–camu seed. The vescalagin and castalagin compounds were extracted, showing high antioxidant, antiproliferative and cytotoxic capacities against A549 and HCT8 cancer cells. It also showed antimicrobial effects, protected human erythrocytes against haemolysis, inhibited α-amylase and α-glucosidase enzymes and presented in vitro antihypertensive effects.

### Enzyme-assisted extraction (EAE)

The enzyme-assisted extraction is usually applied to those bioactive compounds which are not easily extracted through conventional techniques and are tightly bonded in the cell wall. In this technology, the cell wall is hydrolyzed by enzymes, and due to this, the bioactive compounds present inside the cell plasma oozes out from the cell (Nadar et al. 2018; Panja 2018). Some of the most common and frequently used enzymes for this purpose are polygalacturonase, xylanase pectin esterase, polygalacturonase, cellulase, hemicellulase, amylase, b-galactosidase, protease, 1,4-glycosidase, tannase, papainase, and tyrosinase (Barbosa et al. 2020; Liu et al. 2016).

The enzyme plays a significant role in the extraction process. It binds with the cell wall's active site, which is composed of a polysaccharide–lignin network and hydrolyses the polysaccharide and lipid body structures by breaking glycoside bonds in the cell wall and proteolytic bonds in middle lamella (Grassino et al. 2020; Zhang et al. 2019). Thus, the bioactive compounds present inside the cell and middle lamella is extracted through the cell wall. The selection of enzymes for extraction depends on the isolation of target compounds that either degrade the pectin, polysaccharides or break the cell wall to isolate them (Azmir et al. 2013; Lombardelli et al. 2020). Even though this method is better suited than conventional technologies, the cost of enzymes and high extraction time are some disadvantages.

Hamid and Ismail (2020) found that the seed of dates has carbohydrates composed of lignin, cellulose, and hemicelluloses. They examined the waste and showed that lignin and cellulose could be hydrolyzed by enzymes to form fermented sugars with the highest yield of 31 g/L at the optimum condition of 120 FPU/g of enzyme for 6 h, at 45 °C, where the enzyme was taken as cellulase. Similarly, Vasco-Correa and Zapata (2017) reported that the pectin extracted from passion fruit peel using proto-pectinase enzyme has a better yield than the conventional chemical extraction. The pectin was extracted about 40% higher by applying an enzyme of 30 U/ml at pH of 3 and 37 °C. Similarly, the pomegranate seed from the juice industry has a high percentage of fatty acid (22.9%), protein (13.2%), and dietary fibers (97.2%). The enzyme-assisted extraction when using protease enzyme at a concentration of 50 U/g for 14 h, at 45 °C and pH 7.2 is found to have a higher yield of oil recovery (4%) than the Soxhlet extraction (Talekar et al. 2018).

Xu et al. (2014) conducted a study comparing polysaccharide extraction from grape peel using solvent extraction (using ethanol) and with a combination of enzymes, such as cellulase, pectinase, and β-glucosidase. The study reported that the enzyme-assisted extraction required less time for a higher yield of pectin, which contains phenolic compounds, anthocyanin than the solvent extraction. Another study reveals that combining enzyme-assisted extraction and supercritical extraction can be a better technological combination for obtaining a better yield of polyphenols, such as vanillic, ferulic, and syringic acid from pomegranate peels. The mixture of enzymes (pectinase, protease, and cellulase) in the ratio of 25:25:50 at a concentration of 3.8%, temperature 49 °C, time of treatment 85 min and pH of 6.7 caused the highest yield of extract containing phenolic compounds (Mushtaq et al. 2015). Furthermore, the pineapple peel is a source of vinegar in sugar production, which can also be extracted with enzymes, such as cellulase, hemicellulase, and pectinase (Roda et al. 2016).

### Supercritical fluid extraction (SFE)

Due to the degradation of natural thermosensitive compounds, extraction using enzymes and solvents loses popularity among industries. These technologies are now mainly replaced by SFE technology, also known as green technology. It is an advanced form of solvent extraction technology, where different solvents are used as fluid under pressure ranging from about 200 to 400 bar and temperature ranging from 40 to 60 ºC (Gullón et al. 2020; Pinto et al. 2020). Among the solvents, toluene, ethylene, ammonia, and carbon dioxide are primarily used due to their availability, environment safety, non-explosibility, non-toxicity, less expensive, and easily removable properties (Al-Otoom et al. 2014; Vardanega et al. 2019).

The fluid subjected to biomass is in the supercritical condition, where it behaves, such as both liquid and gas. Mainly carbon dioxide is used as the fluid; it is converted to the supercritical stage beyond its critical point by applying a pressure of 7.38 MPa and temperature of 31 ºC (Al-Otoom et al. 2014; Ding et al. 2020). In this condition, the solvent's polarity decreases, and solubility increases, helping the fluid penetrate the cell wall quickly. The bioactive compounds dissolved with this supercritical fluid and extracted along with it (Torres-Ossandón et al. 2018). There are different types of equipment and processes used for SFE, which are described in Table 1. Sometimes, modifiers are used with the fluids to increase their polarity, extracting compounds with high polarity (Gallego et al. 2019).

Cruz et al. (2017) examined and compared the extraction of phenolic compounds from jelly palm seeds with the help of soxhlet extraction, ultrasound-assisted extraction, and SFE with CO_2_ as solvent and ethanol as co-solvent. The study found that the SFE has improved the extraction yield with carbon dioxide, ethanol, and water. Similarly, the SFE was found superior over soxhlet extraction in extracting oil composed of fatty acid and phenolic compounds from guava seed at optimum conditions of temperature 52 °C and 35.7 MPa (density of CO_2_: 895 kg/m^3^) at a constant flow of 30 g CO_2_/ min for 150 min. During extraction, linoleic and oleic acid is found to be the major fatty acid components in guava seed oil. Vanillin, cinnamaldehyde, vanillic acid, cinnamic acid, phytosterols, tocopherols, β-sitosterol, and γ-tocopherol, was the primary phenolic compound found in the oil (Narváez-Cuenca et al. 2020). Cuco et al. (2019) examined that a higher yield of linoleic, oleic, and linolenic acid was obtained when oil was extracted from the pumpkin seed using SFE technology at the optimum conditions of 22 Mpa and 333 K than the ultrasound-assisted extraction (power of 165 W and frequency of 25 kHz) with the solvent as n-hexane. Similarly, the yield of oil obtained from the apple seed was higher through SFE technology than soxhlet extraction. At 24 MPa pressure, 40 °C temperature, 1 L/h of carbon dioxide flow rate and 140 min of treatment time, the yield of oil was 20.5%, where at 4 h of soxhlet extraction the yield of oil was 22% but the difference between both oil was the percentage of unsaturated fatty acid present in it (Ferrentino et al. 2020).

Natolino and Da Porto (2019) explained the extraction of oil, which contains the phenolic compound punicic acid, from pomegranate seed using SFE at 60 ºC and 320 bars. The study also measured the solubility of oil to find out its antioxidant activity. The solubility in SC-CO_2_ is a characteristic property of the sample in the form of fatty acid content and oxygen stability. In the same way, different studies found that SFE using CO_2_ (2 g/ min) was successful in extracting phenolic compounds, such as terpene, γ-element, germacrene, ellagitannins, and flavanols from Brazilian cherry at high pressure and low temperature and the extraction at a pressure at 25 MPa, the temperature at the inlet to the reactor 40 °C, the mass of raw material loaded into the reactor 300.0 g. Flow rate CO_2_ 6.0–7.0 kg caused the higher yield of phenolic compounds from strawberry seeds (e Santos et al. 2015; Grzelak-Błaszczyk et al. 2017). This technology also helped in recovering maximum oil from rosehip seed at the optimum conditions of pressure 30 MPa, the temperature of 40 ºC, concentration 0.75 mL/min, particle size 355 < Dp < 500 µm, and time of 150 min (Salgın et al. 2016). Another study from Marić et al. (2020) suggested that the SFE process at optimum conditions of pressure (300 bar), temperature (50 °C), concentration (0.3 kg CO_2_/h) and treatment time (4 h) is superior over all other soxhlet extraction processes for 8 h in the recovery of oil from raspberry seed which is composed of a high amount of ω-3 fatty acids as well as tocopherols and antioxidants. Furthermore, Gustinelli et al. (2018) also successfully extracted vitamin E containing oil from bilberry seed using SFE with CO2 as the solvent at the optimum conditions of 20 MPa and 60 °C for 80 min.

### Subcritical fluid extraction (SWE)

The modifiers may increase the cost of SFE technology and makes it uneconomical. Hence another method of extraction, SWE, is popular as a replacement for SFE. Here, the fluid is used as a solvent in its subcritical condition between the boiling and critical point (Essien et al. 2020; Zhang et al. 2019). Several polar solvents are used in this process (Table1). However, water is most commonly used among these solvents due to its lower basicity, high polarity, high diffusivity, and environmental safety (Essien et al. 2020).

The polarity and viscosity of water decrease with rising temperature from 100 to 374 ºC and pressure from 1 to 22 MPa. Its solubility increases, which helps extract the bioactive compounds by diffusion, partitioning, equilibrium, or convection. SWE is a cheap and clean technique; however, oxidation and hydrolysis of the pectin chain were reported in a few situations (Gallego et al. 2019; Zhang et al. 2020).

Rodrigues et al. (2019) showed that the extracts from papaya seed could be separated with subcritical water extraction at 150 ºC in 5 min and found that ferulic, mandelic, and vanillic acids are the main components of phenolic groups, which are significantly higher than the soxhlet extraction at 40 °C for the time of 6 h. Another study shows that sweet passion fruit seed oil contains unsaturated fatty acid (86.36%) and tocopherols, potent antioxidants used in the chemical and pharmaceutical industries. This oil extracted using subcritical propane extraction at 60 ºC and 6 MPa pressure provided a significantly higher yield than soxhlet extraction at 65 °C for 4 h, where the solvent was taken n-hexane (Pereira et al. 2017). A similar study on extraction of polyphenols from grape seed and peel using subcritical water extraction in semi-continuous mode reveals that yield in total polyphenol of 44.3 to 77 mg/g and 44 to 124 mg/g from peels and seed, respectively, was obtained when the water was subjected to a temperature of 80–120 °C and pressure of 100 MPa (Duba et al. 2015).

Liew et al. (2018) described pectin extraction from pomelo fruit peel using subcritical water extraction technology in a dynamic mode. During the study, the obtained yield of low methoxyl pectin was 18.8% in the optimized condition of 30 bar pressure and temperature of 120 °C. A similar study on citrus Juno's fruit peel, which contains a high amount of dietary fiber, oils, pectin, hemicelluloses, and cellulose, reveals that 78% of pectin and 80% hemicelluloses and cellulose were separated from the peels after subcritical water extraction in a semi-continuous mode, where the optimum conditions were at a temperature of 200 °C, pressure 20 Mpa in each water flow rate 2.1, 3.5, and 7.0 mL/min (Tanaka et al. 2012).

### Microwave-assisted extraction (MAE)

Despite the risk of oxidation, MAE is one of the most promising technology for obtaining bioactive compounds from fruit by-products. This technology uses microwave energy as a generator of heat, where the samples are subjected directly or indirectly. The microwave spectrum is the electromagnetic wave of combined electric and magnetic fields ranging from 300 MHz to 300 GHz and wavelength from 1 cm to 1 m. However, in MAE, the most frequently used frequency ranges from 915 to 2450 MHz, and wavelength ranges from 12 to 20 cm (Ciriminna et al. 2016; Garavand et al. 2019).

The microwave radiation applied to the sample generates heat by rotation and displacement of ions and molecules, which causes localized heating from inside to outside (Maran et al. 2015; Mena-García et al. 2019). Dipole rotation and ionic conduction are the leading cause of mass and heat transfer from cell to cell matrix. The bonds between tissues and molecules are broken by water evaporation, causing the extraction of volatile and non-volatile compounds. Upon microwave treatment, the cell wall ruptures so that the bioactive compounds present inside the cell wall can penetrate out of the cell (Adetunji et al. 2017). The moisture present in the biomass and microwave power causes rapid heating and rapid extraction of compounds from the product (Mena-García et al. 2019).

Al-Dhabi and Ponmurugan (2020) proved that the Jamun seed contains a high amount of polysaccharides which can be extracted through MAE. In the study, the polysaccharides yield obtained 4.71% at a microwave power of 515 W, pH of 3.2, and time of 3.1 min, and SL ratio of 1:15 g/ml. Similarly, Torres-León et al. (2017) showed that the high amount of bioactive compounds from mango seed could be extracted through MAE at optimum conditions of solid to solvent ratio 1/60 g/mL, treatment temperature of 75 ºC, and extraction cycle of 2 cycles, where the solvent was taken as ethanol. The study also reported that the primary antioxidants found in mango seed are ethyl gallate, pent-*O*-galloyl-glucoside (PGG), and hamnetin-3-[6-2-butenoil-hexoside]. Furthermore, the dragon fruit peel contains a methoxyl group of pectin which can be extracted maximum using MAE with an operating condition of 450 W power and 5 min, then CE processes at 85 °C for 1 h treatment time. The solvent was taken as water mixed with 0.05 M of nitric acid (Tongkham et al. 2017).

Avocado seeds are agro-industrial residues that can be used as the source of antioxidants, phenolic acids, procyanidins dimer B, catechin, epicatechin. Araújo et al. (2020) showed that a maximum amount of bioactive compounds could be extracted from avocado seeds using microwave-assisted solvent extraction with two different solvents, acetone (72.18 °C and 19.01 min) and ethanol (71.64 °C and 14.69 min), respectively. Likewise, watermelon peel contains a considerable amount of pectin, which can be extracted with maximum yield through MAE at a microwave power of 477 W, the irradiation time of 128 s, pH of 1.52, and a solid/liquid ratio of 1:20 g/mL, where the distilled water was taken as solvent (Maran et al. 2014).

The pineapple peel is a good source of pectin and polyphenolic compounds, which can be extracted through MAE at a power of 420 W and irradiation time of 30 min, compared with CE done for 60 min (Rodsamran and Sothornvit 2019b). Similarly, the peel of mahkota dewa, a well-known medicinal plant, is composed of phenolic compounds which can be successfully extracted using MAE technology with a yield of 61.25% at a microwave power of 300 W in 1 min at 80 °C, where the solvent was taken as water and solid to solvent ratio was 60 g/ml (Alara et al. 2019).

### Ultrasound-assisted extraction (UAE)

UAE uses the sound energy produced by ultrasonic waves with frequencies ranging from 20 to 100 kHz (Belwal et al. 2020; Dzah et al. 2020; Saifullah et al. 2020). Because of its advantages, this emerging technology is also called green technology. The major drawback of this process is the number of repetitions required to complete the extraction process, resulting in a lot of time and energy consumption (Carrillo-Hormaza et al. 2020; Chakraborty et al. 2020).

The cavitation is the leading cause of extraction, which is generally generated by the ultrasonication process ranging from 20 to 40 kHz. Cavitation is the phenomenon in which the microbubbles are formed, enlarged, and imploded. The generation of microbubbles is caused by ultrasonic waves of continuous compression and the rarefaction process. This cavitation helps in the mass transfer process between the liquid extracting medium and the solid plant materials for enhancing the extraction process (Alirezalu et al. 2020; Grassino et al. 2020; Meregalli et al. 2020).

During UAE, the negative pressure formed in rarefaction causes cavitation, leading to the formation of bubbles that collapses near the solid materials. Localized heating due to the high speed of the liquid jet causes fragmentation of brittle materials. The desired compounds are eroded from the plant cell when the cell gets ruptured by turgor pressure created by the cell wall because of the solvent medium's entry (Chen et al. 2019; Machado et al. 2019; Ojha et al. 2020). Different mechanisms are responsible independently for the rupturing of the cell wall, such as fragmentation, erosion, sonoporation, sono-capillary effect, local shear stress, and detexturization (Chemat et al. 2017).

Rambutan fruit peel can be a source of polysaccharides that could be extracted through the UAE. Maran and Priya (2014) proved that the rambutan fruit peel could be a good source of polysaccharides which can be extracted with its maximum yield using the UAE technique at the optimum liquid to solid ratio of 32:1 ml:g, ultrasonic power of 110 W, extraction temperature of 53 °C and extraction time of 41 min. Similarly, this technology successfully extracted the polysaccharides from the jujube seeds at the optimum temperature of 83.1 °C, time of 100 min, ultrasonic power of 140 W, and water-material ratio 33.5 mL/g, which have antitumor activity (Wu et al. 2019). Likewise, UAE was found superior to conventional and soxhlet extraction technologies for extracting the bioactive compounds with higher antioxidant activity (1.2 times higher) from Jamun seed powder at the optimal condition of 12 min extraction time, solid to water ratio 1:15, temperature 35 °C, power 125 W, and duty cycle 60% (Mahindrakar and Rathod 2020).

Another study on baobab seed which has a high amount of phenolic compounds reported that the UAE process at 20 min, 30% amplitude, 60 °C temperature, and 30 ml/g solvent to solid ratio results in an extract that contains a high amount of flavonoid contents and antioxidant activity than the conventional solvent extraction (Ismail et al. 2019). A similar study by Raj and Dash (2020) described that the UAE is better among other technologies in extracting pectin from dragon peel. The extract contained a higher yield of phenolic compounds, antioxidants, and betacyanin of the optimum conditions at an ultrasonic temperature of 60 °C, solvent to solid ratio 25:1 mL/g, solvent concentration 60%, and ultrasonic treatment time of 20 min. Moreover, the highest amount of total phenolic content (1493.01 mg GAE/100 g) was extracted from mango peels using UAE technology because of higher ultrasound intensity. The solvent was taken as a blend of ethanol-acetone (Martínez-Ramos et al. 2020).

Furthermore, maximum polysaccharides yield of 15.94% was obtained from canister seed when UAE was applied at an ultrasonic temperature of 79 °C, ultrasonic time of 69 min, and liquid to the material ratio of 41 ml/g, where the solvent was taken as water (Ma et al. 2020). Similarly, the strawberry peel is rich in bioactive compounds beneficial to human health, such as phenolic compounds, carotenoids, flavonoids, and anthocyanins which can be extracted by the UAE technique. The anthocyanin recovered from the peel was 12% more in UAE at optimum conditions of time 90 min and frequency of 50 kHz than the CE process (maceration) at time of 120 min, with constant ethanol concentration (90%), pH 1.5, and sample mass/solvent volume ratio (1 g/10 ml) (Meregalli et al. 2020).

### Pulsed-electric field-assisted extraction (PEFAE)

The PEFAE can be considered a novel extraction technology for bioactive compound extraction due to purity, less energy requirement, and environment-friendly solvent usage. It is also called a non-thermal extraction process, because the natural compounds are recovered at a minimal temperature without losing quality and nutritional value (Andreou et al. 2020; El Kantar et al. 2018a). Electroporation is the primary mechanism behind pulsed electric field extraction. In the process of PEFAE, the electrical energy is applied for the creation of nano/micro-poration of the cell membrane so that the bioactive compounds present inside the cell plasma could extract out of it (Puértolas et al. 2013; Shorstkii et al. 2020). The electric pulses cause the transfer of ions and molecules from inside the cell towards the cell membrane (phospholipids bounded molecule), acting as an insulator (Plazzotta et al. 2021).

In a study, the apple peels were treated under the pulsed electric field with different electric intensities and times to extract the phenolic compounds. The extraction is analyzed concerning the electrical conductivity (disintegration index) and confocal laser scanning microscopy (microscopic cell disintegration index). The results reported that the extraction was dependent on the cell integration index and the electric field intensity. The study also confirmed that the higher the intensity (1200 V/cm) at constant cell integration constant, the higher the soluble matter recovery (Wang et al. 2020). Similarly, Parniakov et al. (2015) reported the extraction of bioactive compounds from papaya seeds by PEFAE. Here, the recovery was influenced by undesirable chemicals and free radicals when the extraction was done by high voltage electric discharge (HVED). The extract obtained from PEFAE along with solvent aqueous extraction about 50 °C, pH-7 and time of 5 h had a higher yield (200%) containing 20% higher antioxidant capacity than CE. Likewise, pear peel is a good source of red colour compounds, such as betanin and isobetanin, which can replace the synthetic colourings in the industry. Koubaa et al. (2016) proved that the maximum yield of colour has happened during the PEFAE at the intensity of 20 kV/cm along with 300 pulses followed by supplementary aqueous extraction of 1 h as compared to the conventional grinding followed by supplementary aqueous extraction of 24 h.

Another study by Parniakov et al. (2016) showed that the yield of antioxidants, protein, and carbohydrates from the mango peels was maximum when PEFAE at intensity 13.3 kV/cm was combined with aqueous extraction at 50 °C temperature for 5 h at six pH, which was found higher than the aqueous extraction at temperature 60 °C and pH 6. However, the yield was comparatively less when extracted separately using PEFAE or using aqueous extraction. Similarly, Luengo et al. (2013) conveyed that an electric field treatment of intensity 7 kV/cm with a pulse rate of 20 in 60 microseconds was found successful in extracting the polyphenols and flavonoid compounds from orange peel.

## Combination of extraction (CE) process

The extraction technologies mentioned above can also be used in combinations to further reduce the extraction time, increase the extraction yield, and overcome the limitations of the single technologies (Zuin and Ramin 2018). The pre-treatment of fruit by-products using the microwave or ultrasound or enzyme before any CE process for the rapid breakdown of cell walls is one of the commonly followed combined extraction treatments. Similarly, two or more emerging extraction processes can also be used in combinations, such as SFE and UAE, MAE and solvent extraction, enzyme application before treatment of PEF, and supercritical fluid treatment before MAE followed by supplementary solvent extraction and so on (Dias et al. 2019). A few examples of combined extraction processes for bioactive compounds recovery from fruit by-products are summarized in Table 3.

**Table 3:** Combination of extraction technologies for the recovery of bioactive compounds from fruit by-products

Sl. No.	Name of the by-product	Combination of extraction processes	Optimized condition	Bioactive compounds	References
1	*Vitis vinifera* (Grape) peel	UAE + PEFFAE	Ultrasonic energy with 50 °C with a pulse of flow 290 L/h, diameter of chamber 25 mm, gap 26 mm, 25 kV voltage	Anthocyanin Flavonoids	(Medina-Meza and Barbosa-Cánovas 2015)
2	*Litchi chinensis* (Litchi) seed	UAE + PEFAE	30% aqueous ethanol, 62.66 mL/g ratio of liquid: solid, 123 mL/min flow velocity, 276 W ultrasonic power, 47 °C ultrasonic temperature, discharge voltage 14 kV	Saponins	(Fan et al. 2020)
3	*Citrus aurantiifolia* (Lime) peel	MAE + UAE	Microwave power 140 W with 55% ethanol 45 s and ultrasonic energy of 38% amplitude for 4 min	Phenolic compounds, Antioxidants	(Rodsamran and Sothornvit 2019a)
4	*Passiflora edulis* (Passion) seed	SPE + UAE	Ultrasonic power of 160 W and at 40 °C and pressure of 16 MPa in SFE	Tocopherols Tocotrienol	(Barrales et al. 2015)
5	*Citrus sinensis* (Orange) peel	EAE + PEFAE	The high voltage of energy input of 222 kJ/kg with enzymatic hydrolysis viscozyme of 12FBGU/g	Polyphenols, Reducing sugars	(El Kantar et al. 2018b)
6	*Punica granatum* (Pomegranate) seed	SPE + MAE + CE	Microwave radiation of 250 W with 6 min and then by SFE and Soxhlet extraction	Punicic acid	(Đurđević et al. 2017)
7	*Spondias tuberose* (Umbu) seed	SPE + UAE	SFE at 40 °C with 15 Mpa and then ultrasonic power of 500 W with 4 min and 1:30 S/L ratio	Free fatty acid, Phenolic compounds	(Dias et al. 2019)
8	*Litchi chinensis* (Litchi) peel	MAE + UAE	MAE in 70 °C in 4 min of extraction with 40:1 solvent: material ratio and UAE in 3 min of extraction and chlorobenzene as a solvent	Pyrethroid	(Wang et al. 2018)

## Conclusion

This review describes both conventional and emerging techniques used to extract bioactive compounds from the by-products of fruit processing industries. The primary process parameters, advantages, disadvantages, and applications of each technology were explained in detail. Moreover, the bioactive compounds extracted from extraction technologies were characterized in chromatographic and spectrophotometric methods. With an increase in the number of fruit processing industries, the number of by-products from these industries also increases. Management of these by-products is a considerable burden for the industries, and the improper disposal of these by-products can create significant harm to the environment. Hence, its utilization as a source of bioactive compounds will increase industries' financial status and decrease their burden of waste management. Improvement in extraction technology with lesser or no use of solvents will significantly impact a sustainable bioprocess. In addition, the characterization of bioactive compounds by rapid, sensitive, and cost-effective methods helps for the utilization and incorporation in the various field. Industrial by-products can be converted into a potential source of bioactive compounds. The compounds extracted from it can be incorporated into various products in the pharmaceutical and food industries.

## Data Availability

Not applicable.

## Abbreviations

CE: Conventional extraction
EAE: Enzyme-assisted extraction
SFE: Supercritical fluid-assisted extraction
SWE: Subcritical water-assisted extraction
MAE: Microwave-assisted extraction
UAE: Ultrasound-assisted extraction
PEFAE: Pulsed electric field-assisted extraction
DPPH: 2,2-Diphenyl-1-picryl-hydrazyl-hydrate
ABTS: 2,2'-Azino-bis (3-ethylbenzothiazoline-6-sulfonic acid)
FRAP: Ferric reducing ability of plasma
ORAC: Oxygen Radical Absorbance Capacity
HPLC: High-pressure liquid chromatography
TLC: Thin liquid chromatography
GC: Gas chromatography
MS: Mass chromatography
MIC: Minimum inhibitory concentrations
DAD: Diode-Array Detection
ESI: Electrospray ionization
